# MVSAPNet: A Multivariate Data-Driven Method for Detecting Disc Cutter Wear States in Composite Strata Shield Tunneling

**DOI:** 10.3390/s25061650

**Published:** 2025-03-07

**Authors:** Yewei Xiong, Xinwen Gao, Dahua Ye

**Affiliations:** 1 SHU-SUCG Research Centre of Building Information, Shanghai University, Shanghai 201400, China; xiongyewei@shu.edu.cn (Y.X.);; 2 School of Mechatronic Engineering and Automation, Shanghai University, Shanghai 200444, China

**Keywords:** tunnel boring machine, disc cutter, deep learning, interpretability, time series classification

## Abstract

Disc cutters are essential for shield tunnel construction, and monitoring their wear is vital for safety and efficiency. Due to their position in the soil silo, it is more challenging to observe the wear of disc cutters directly, making accurate and efficient detection a technical challenge. However, existing methods that treat the problem as a classification task often overlook the issue of data imbalance. To solve these problems, this paper proposes an end-to-end detection method for disc cutter wear state called the Multivariate Selective Attention Prototype Network (MVSAPNet). The method introduces an attention prototype network for variable selection, which selects important features from many input parameters using a specialized variable selection network. To address the problem of imbalance in the wear data, a prototype network is used to learn the centers of the normal and wear state classes, and the detection of the wear state is achieved by detecting high-dimensional features and comparing their distances to the class centers. The method performs better on the data collected from the Ma Wan Cross-Sea Tunnel project in Shenzhen, China, with an accuracy of 0.9187 and an F1 score of 0.8978, yielding higher values than the experimental results of other classification models.

## 1. Introduction

The shield machine, as a kind of tunnel driving equipment with fast tunneling speed, high geological adaptability, high safety, and minimal impact on the construction ground, has become the primary method for modern tunnel construction [1]. As the core component in the tunneling process, disc cutters cut broken rock with rotation of the cutterhead and gradually wear out. When the amount of wear accumulates to a certain extent, the overall cutting performance of the shield will decrease. If disc cutters are not replaced in time, other components, even the whole cutterhead, can be further damaged, seriously affecting the construction progress and the service life of the shield machine. Currently, the methodologies for assessing disc cutter wear can be categorized primarily into three approaches: periodic inspection, direct assessment with sensor-based monitoring [2,3,4], and indirect assessment through data analysis [5].

Although regular inspection is the most effective and direct method, it has some problems such as the need to shutdown, not being monitored in real-time, reducing the efficiency of excavation, and producing safety hazards with frequent open warehouses. According to statistics, in some tunneling projects, regular warehouse inspection even consumes about one-third of the construction cycle time [6]. The sensor-based method has the advantages of real-time detection and noncontact. However, the location of sensors in actual propulsion is often under high temperature and pressure conditions, which is prone to failures and performance degradation. In addition, there are also problems of time consumption and cost in sensor placement and replacement, and it is difficult to achieve full real-time detection in actual use [7].

Unlike regular inspection and the sensor-based method, the indirect assessment method utilizes the existing data from tunnel construction for calculation or fitting to estimate the amount and state of wear indirectly. It mainly consists of two kinds: the mechanism method and the data-driven method. The mechanism-based method establishes mathematical expressions that correlate characteristic parameters influencing disc cutter wear with wear indicators by examining the rock-breaking process and motion patterns of disc cutters, in conjunction with the mechanisms of wear [8,9,10]. While the mechanism-based analytical method can accurately estimate the wear of individual cutters under normal excavation conditions in specific environments and determine the need for cutter replacement, its effectiveness in detecting cutterhead wear is limited in cases of abnormal wear [8] or complex strata with changing geological properties [11,12,13].

Fortunately, with the proven capability of machine learning and deep learning in handling complex, interdependent data across various domains, constructing network models for detecting disc cutter wear using data-driven approaches integrated with operational parameters has become feasible. Liu et al. [7] argue that during excavation, monitoring the condition of each blade is generally unnecessary. The overall cutting performance of the cutterhead is considered more important than the wear value of individual blades and is thus treated as a binary classification problem. However, due to the typically limited number of wear samples, which are much fewer than normal samples, data imbalance inevitably arises. This imbalance can degrade network training performance and reduce generalizability for minority class samples. To address this, the objectives of this study are the following: (a) Develop a data-driven deep learning model to classify cutterhead wear status in shield machines by integrating operational sensor data from routine tunneling operations. (b) Overcome the limitations of small sample sizes and data imbalance while maintaining superior classification performance. (c) Ensure interpretability to elucidate the influence of input parameters on classification outcomes. To achieve these objectives, this paper proposes a Multivariate Selective Attention Prototype Network (MVSAPNet). Utilizing operational data from shield tunneling projects, after preprocessing steps to select features strongly correlated with wear, the proposed model is used for training to classify disc cutter wear. The main contributions of this paper are as follows:

(1) We propose a new network, the Multivariate Selective Attention Prototype Network (MVSAPNet), which introduces a prototype learning approach to tunnel wear state classification, **addressing class imbalance and small sample sizes**.
(2) A variable selection network is used to select input features, **enhancing the model’s nonlinearity and interpretability** while providing insights into the classification results.
(3) The model is tested on tunneling data from composite strata in tunnels located in Shenzhen, Guangdong Province, and Fuzhou, Fujian Province, China, **demonstrating superior performance compared to other classification models**.

The rest of this paper is arranged as follows. Section 2 reviews existing data-driven approaches for disc cutter wear detection, highlights their shortcomings, and introduces the method proposed in this paper. Section 3 introduces the preprocessing of historical data features, feature extraction, and the proposed MSVAPNet method. Section 4 uses historical data from two tunnels in Shenzhen and Fuzhou for training and testing and compares the performance of the proposed method with other approaches. Section 5 analyzes and discusses the network model used in the study. Finally, Section 6 provides our conclusion.

## 2. The Literature Review

### 2.1. Overview

Existing data-driven approaches for disc cutter wear detection primarily adopt regression or classification paradigms.

Regression-based methods [14,15,16,17] predict wear levels or introduce synthetic indicators, but they exhibit limitations such as geological changes, abnormal wear conditions, and the requirement of frequent recalibration.

Unsupervised classification approaches [18] leverage LSTM-ED to capture patterns through normal operational data, employing threshold-based health assessments to detect the disc cutter wear. While effective for detecting wear degradation, these methods risk misclassifying nonwear anomalies like clogging or shield adjustments.

The detection of disc cutter wear can also be framed as a supervised classification problem [7,19]. The supervised method not only improves the detection of abnormal wear but also reduces false alarms compared to unsupervised approaches.

Currently, the extant methodologies still exhibit the following issues.

### 2.2. Data Imbalance

Supervised classification methods seem to be relatively better. However, a challenge arises due to the typically much smaller number of wear samples compared to normal ones, resulting in data imbalance that affects model performance and generalization. Liu et al. [7] balanced the class distribution by sampling normal samples, though this may overlook important data features. Qin et al. [20] used a k-means-SMOTE algorithm to augment clogging samples, which address imbalances but can create marginal synthesized samples and blur classification boundaries. Since cutter wear is a gradual process and the boundary distinction is not clear enough, the SMOTE may further obscure these boundaries, complicating model training and making it less effective for wear detection.

To solve this problem, we introduce the prototype network method used in few-shot learning, which is widely used in multivariate time series classification tasks [21,22,23]. This approach addresses class imbalance by representing each class through characteristic prototypes and classifying instances via prototype distance metrics, making it well suited for shield cutter wear detection.

### 2.3. Deep Learning

In addition, although machine learning methods such as SVM and Random Forests have been widely used in shield cutter wear detection, they inevitably have certain limitations, such as a lack of feature extraction capability, overfitting, and high computational complexity [17,24]. With the continuous development of deep learning methods in recent years, the ability to model and fit data has significantly improved rapidly. At the same time, the number of input parameters involved increases; some studies on cutter wear detection, for example, consider up to 10 or more parameters [7,14]. In the context of high-dimensional data, traditional machine learning methods are increasingly being surpassed by deep learning approaches in terms of accuracy. Zhang et al. [14] proposed a 1D CNN integrated with a GRU network to predict cutter life, demonstrating significantly higher prediction accuracy compared to various machine learning algorithms, such as ExtraTrees, Gradient Boosting Machine, Random Forest, AdaBoost, and k-NN.

Consequently, to improve both the performance and the efficacy of the model, this paper adopts deep learning methods for detection and introduces a variable selection network to model spatial relationships between variables. This not only augments the model’s capacity to capture nonlinear patterns but also provides a clear visualization of the contributions of different input features to the model’s overall prediction, thereby improving interpretability. In addition, LSTM networks are incorporated to capture temporal dependencies within the input data. Finally, an attention-based prototype network is used to address the classification and detection of disc cutter wear in small-sample, imbalanced scenarios.

## 3. Materials and Methods

**Figure 1:**
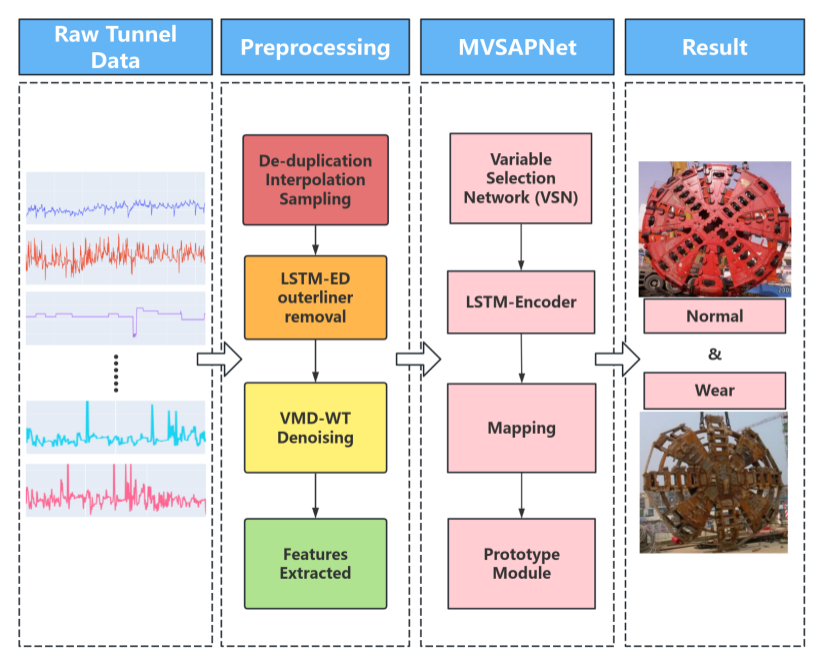
The overall framework of the disc cutter wear detection.

This section is primarily composed of different parts, which is shown in Figure 1. First, for the raw data extracted from the database, due to the presence of a large amount of data during the assembling stage that are unrelated to the wear process, it was necessary to extract the data from the advanced state and then reconstruct them to remove outliers using the LSTM-ED algorithm [25], followed by denoising with the VMD-WT algorithm. Afterward, the data features were initially extracted through feature engineering using a specific method and then split into datasets. Finally, the data were trained using the classification detection model proposed in this paper to classify the disc cutter’s state.

### 3.1. Preprocessing

**Figure 2:**
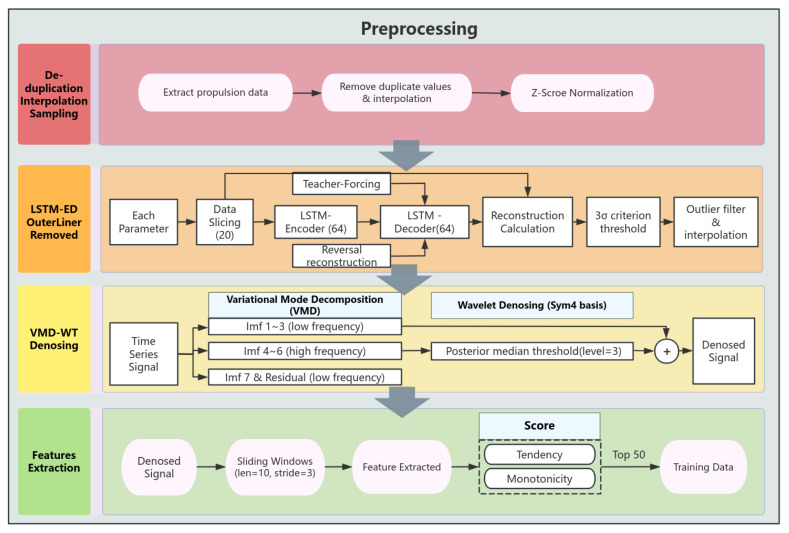
The architecture of data preprocessing.

The acquired shield operation data require a series of preprocessing steps to make the original sensor data more suitable for training. The specific process is shown in Figure 2. First, shield tunneling is divided into two states: the propulsion state and the assembling state. Since the disc cutter primarily wears out during the propulsion state, it is necessary to extract the data corresponding to the shield’s propulsion state from the database. For the acquired propulsion state data, the Z-Score method is applied using the following formula:

(1) $${\hat{x}}_{t}^{i}=\frac{x_{t}^{i}-\mu^{i}}{\theta^{i}},\left(0<i\leq N\right)$$

where ${\hat{x}}_{t}^{i}$ denotes the normalized data of $i^{th}$ parameter at the moment *t*. $x_{t}^{i}$ denotes the raw data, $\mu^{i}$ represents the mean value, $\theta^{i}$ represents the standard deviation, and *N* denotes the number of selected sensor data.

In addition, due to various disturbances and abnormalities during the shield tunneling process, raw data often contain numerous outliers. Therefore, it is essential to detect and eliminate these outliers. Commonly used outlier detection methods include mean square deviation, box plot, clustering, reconstruction, and others. Autoencoder (AE) is an unsupervised learning method designed for data reconstruction and feature learning that is commonly used for dimensionality reduction or anomaly detection. It primarily consists of two components: an encoder, which compresses data into a latent space, and a decoder, which reconstructs the data from that latent space. The autoencoder can accurately reconstruct normal data by learning their feature patterns, but it performs poorly on outliers, making it suitable for anomaly detection. Meanwhile, since LSTM can extract feature information from time series, it is better at capturing long-term dependencies in time series compared to CNN architectures. Additionally, LSTM addresses the gradient vanishing and gradient explosion issues commonly encountered by traditional RNNs when processing long-sequence data, making it more suitable for encoding shield tunneling time series data. In this paper, we used the LSTM-ED model [25] to remove outliers from the original data. Additionally, the LSTM-ED model was optimized using inverse-order reconstruction and teacher-forcing strategies, which help to mitigate error accumulation within the model and further enhance its reconstruction capability. The average absolute error was employed as the reconstruction error of the model and calculated using the following formula:

(2) $$l=∥x_{recon}-{x∥}_{2}$$

For the reconstruction error, the outliner is identified using the 3σ criterion, with the threshold defined as follows:

(3) $$Threshod=\mu_{l}+3\sigma_{l}$$

where Threshod denotes the filtering threshold, and $\mu_{l}$ and $\sigma_{l}$ represent the mean and standard deviation of the reconstruction error, respectively. For the average reconstruction error at each time step corresponding to different sliding windows, the threshold is applied to identify outliers. Any identified outliers are discarded and replaced through interpolation.

Although the LSTM-ED method can effectively remove anomalous data collected by shield sensors that deviate from the global distribution, as well as some high-frequency noise, the data collected during shield tunneling are often influenced by various interferences, including those from the equipment sensors themselves, the geological strata, and personnel operations. Consequently, the ability of the reconstruction method alone to mitigate the impact of noise is limited. Therefore, further denoising of the data is necessary. The core concept of Variational Modal Decomposition (VMD) [26] is to decompose a signal into multiple intrinsic modal functions (IMFs) and a residual component. Each IMF represents a distinct frequency component of the signal, while the residual captures the remaining portion of the original signal. Unlike the Empirical Mode Decomposition (EMD) method, VMD addresses the endpoint effect and modal aliasing issues present in EMD by adaptively matching each mode’s optimal center frequency and finite bandwidth. This capability makes VMD particularly effective in reducing the complexity and nonlinearity of nonstationary time series, and it is widely applied across various engineering fields [27,28,29]. Therefore, this paper used VMD combined with WT to denoise and filter the shield data. Using VMD, the shield parameters were decomposed into seven different IMFs, denoted as IMF1–IMF7, with IMF7 having the highest center frequency. For IMF4–IMF6, which contain a certain level of noise, each component was denoised using the WT wavelet transform. The sym4 wavelet was used as the wavelet base, and the noise was removed using empirical Bayes combined with a posterior mean threshold. As for IMF1–IMF3, they were left unchanged due to their lower frequencies, which represent the characteristics of the original signal. Finally, the denoised signal was obtained by summing IMF1–IMF3 and the denoised IMF4–IMF6.

For the denoised shield data, features can be initially extracted using feature engineering to enhance the input to the model. Since the shield machine sensors collect data at a low frequency, typically in seconds, minutes, or in millimeter travel (mm), it is not suitable to analyze the shield timing data from a frequency domain perspective, as this would lead to more serious aliasing problems. Therefore, it is necessary to use sliding windows to construct time domain features in order to analyze the shield data. In line with the features used in other studies [20,30,31], the features selected in this paper are shown in Table 1, where *N* is the window length, and $x_{i}$ represents the denoised data at position *i* within the window. Some formulas were further calculated based on the results of the former formula in the table.

However, when more parameters are selected, time domain feature extraction can lead to a large input dimension, which may result in the “dimensional disaster”. This can introduce noise, leading to overfitting, and deteriorate the generalization ability of the model. Additionally, it increases the complexity of computation and the difficulty of training the model. Therefore, it is necessary to introduce certain indices to the input features in order to reduce the dimensionality of the model. Since disc cutter wear is irreversible, the input features should exhibit a certain degree of monotonicity and trend throughout the complete wear process [32,33]. These metrics can be calculated as follows:

(4) $$\mathrm{Mon}\left(X\right)=\frac{1}{N-1}\left|\sum_{t}\delta \left(x_{t+1}-x_{t}\right)-\sum_{t}\delta \left(x_{t}-x_{t+1}\right)\right|,0<t\leq N-1$$

(5) $$\mathrm{Tred}\left(X,T\right)=\frac{|N\sum_{t}x_{t}t-\sum_{t}x_{t}\sum_{t}t|}{\sqrt{\left[N\sum_{t}x_{t}^{2}-{\left(\sum_{t}x_{t}\right)}^{2}\right]\left[N\sum_{t}t^{2}-{\left(\sum_{t}t\right)}^{2}\right]}},0<t\leq N$$

where $x_{t}$ denotes the data at time *t*, *N* denotes the number of data, δ(·) denotes the step function. After calculating all the data monotonicity and trend metrics, each metric is normalized and then summed to obtain the final score. The features with the top 50 scores are selected as the model inputs for training.

### 3.2. Multivariate Selective Attention Prototype Network

**Figure 3:**
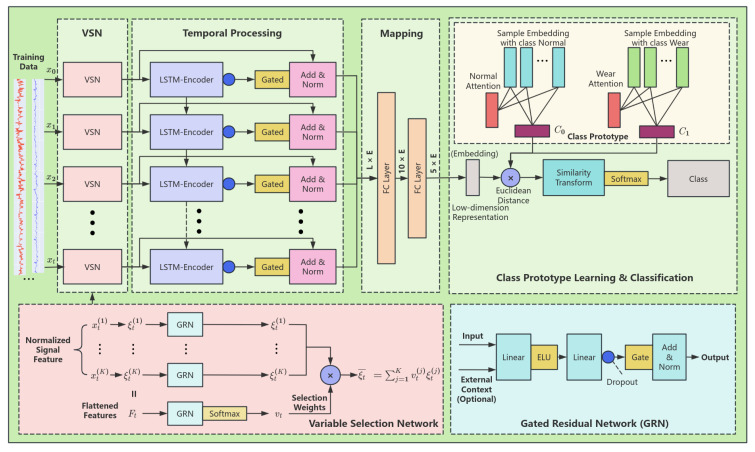
The architecture of MVSAPNet.

After selecting the appropriate features as model inputs, the next step is training using the Multivariate Selected Prototype Classification Network (MVSAPNet) proposed in this paper. Figure 3 shows the overall structure of the model, which consists of three parts: the variable selection network (VSN), Temporal Processing, and Class Prototype Learning.

#### 3.2.1. Variable Selection Network (VSN)

The operating parameters during shield propulsion are influenced by various factors, such as the stratum and the parameters of the tunnel shield itself. These parameters may have a positive effect on the overall training of the model. Inspired by Bryam Lim et al. [34], we introduced the gated residual network (***GRN***) to promote the extracted features, allowing them to better fuse nonlinearly with the auxiliary feature parameters. The overall structure of the ***GRN*** is shown in Figure 3. The ***GRN*** accepts a primary feature and an optional auxiliary context feature as inputs. Afterward, two linear layers are used to initially fuse the two features, and an exponential linear unit activation function (***ELU***) [35] is applied between the linear layers to enhance the fitting ability between the inputs. The results are then input into gated linear units (***GLU***) after a dropout layer, enabling the ***GRN*** to control the degree of contribution from the input feature sequences. Finally, the outputs are superimposed with the main features using residual concatenation, which increases the speed of model training while enhancing the model’s fitting ability.

(6) $$\mathrm{GRN}\left(i,e\right)=\mathrm{Norm}(i+\mathrm{GLU}\left(\eta_{1}\right))$$

(7) $$\mathrm{GLU}\left(\eta_{1}\right)=\sigma \left(W_{1}\eta_{1}+b_{1}\right)⊙\left(W_{2}\eta_{1}+b_{2}\right)$$

(8) $$\eta_{1}=W_{3}\mathrm{ELU}\left(W_{4}i+W_{5}e+b_{4}\right)+b_{3}$$

where *i* denotes the primary feature of input, *e* denotes an optional auxiliary context feature, σ denotes the sigmoid activation function, and *W* and *b* are weights and bias values.

For many input features, conventional classification algorithms find it difficult to accurately measure the specific contributions of different variables to the output. The effectiveness of the model is often evaluated by comparing the features in the middle, which is not an ideal way to show the contribution of different input features to the overall model. To address this, the feature variable selection network (VSN) was introduced as the first part of the model. For a set of input feature sequences, the VSN first encodes each dimension of the input feature data, with the encoding dimension set to 128. This encoding amplifies the input features and represents deeper information within the sequences, making it easier to extract and classify features in the subsequent stages. Afterward, the data for each timestamp are passed into the GRN network, and the data for all timestamps are flattened before being input into the GRN. The corresponding selection weights for each variable are then obtained through a softmax operation. Finally, the feature output is superimposed.

The purpose of introducing the VSN in this way is twofold: it not only enables the selection of input variables but also allows control over the degree of contribution by discarding input or noise that does not significantly contribute to the classification model. This process helps the variable selection network to enhance the overall performance of the classification model.

#### 3.2.2. Temporal Processing

Due to the nature of time series data collected from tunnel machines, analyzing data at a single timestamp is insufficient for making judgments. The real significance of time series data emerges when they are compared across different timestamps in context. Therefore, modeling temporal dependencies is crucial for many time series tasks. Unlike CNNs, which focus more on local patterns, LSTMs are designed with four different gates that allow them to focus on long-term dependencies while retaining short-term information. This ability makes LSTMs particularly well suited for capturing the long-term dependency features inherent in time series data.

In this model, we used an LSTM-Encoder structure to learn the temporal dependencies of the shield feature data. After feeding the data into the LSTM-Encoder at each time step, a corresponding latent layer output is generated. This output is then passed through a gated network and a residual structure, ultimately providing features that capture both the short-term and long-term temporal patterns in the data. This approach ensures that the temporal relationships in the data are effectively learned and used for improved model performance.

#### 3.2.3. Class Prototype Learning

Since the data on disc cutter wear exhibit specific characteristics such as class imbalance and the wear samples being severely limited, conventional classification and detection algorithms face difficulties in training the model and learning the relevant fault features directly. To address this challenge, we drew inspiration from the TapNet model [36] and introduced the attention prototype model into the training process. This model allows for the learning of different class feature prototypes, which are then used to detect the blade wear state by comparing the distance between the test data features and each class prototype.

Figure 3 illustrates the overall structure of the attention prototype model. The process begins with the input features being passed through a mapping layer consisting of two fully connected layers. This mapping layer performs dimensionality reduction on the input features to streamline the learning process, making it easier to distinguish between different classes. The attention mechanism is then applied to these reduced features, focusing on the most relevant aspects of the data and improving the model’s ability to learn from limited wear samples. By comparing the distance between the test data features and the class prototypes, the model can accurately detect the wear state of the disc cutter. Assuming that the input features are $H^{L\times E}\in R^{L\times E}$, where *L* is the length of the sliding window and *E* is the embedding of series data, we have the following:

(9) $$H_{1}^{LE}=\mathrm{Flatten}\left(H^{L\times E}\right)$$

(10) $$H_{2}^{10E}=\mathrm{Relu}\left(\mathrm{BN}\left(W_{1}H_{1}^{LE}+b_{1}\right)\right)$$

(11) $$H_{3}^{5E}=\mathrm{RELU}\left(\mathrm{BN}\left(W_{2}H_{2}^{10E}+b_{2}\right)\right)$$

where Flatten(x) denotes the spreading operation, *W* and *b* are the weight and bias, BN(x) is the batch normalization, and Relu(x) is the activation function.

Afterwards, the dimensionality-reduced data are fed into an attention pool for training based on the labels. Each label corresponds to a different attention model in the attention pool, with the number of classes matching the number of attention models in the pool. Afterwards, an attention score is obtained:

(12) $$A_{k,i}=\mathrm{softmax}\left(W_{k,i}^{T}\tanh\left(V_{k,i}H_{k,i}^{T}\right)\right)$$

where $A_{k,i}$ denotes the weight of *i* sample belonging to *k* class, $H_{k,i}$ denotes the embeddings of input data, $W_{k,i}^{T}\in R^{u\times 1}$ and $V_{k,i}\in R^{u\times d}$ are weights of attention model, and *u* is a settable hyperparameter. Then, we multiply the feature data and the attention score to produce the class vector.

(13) $$c_{k}=\sum_{i}A_{k,i}\cdot H_{k,i}$$

After training, each class will have a corresponding class prototype vector, and these vectors are concatenated to form a class prototype matrix. During the testing phase, the latent features are obtained through the mapping layer, and the distance to each class prototype matrix is calculated. The distance can be measured using methods such as Euclidean distance, Mahalanobis distance, and others. In this paper, we used Euclidean distance $D\left(z,z^{\prime }\right)={∥z-z^{\prime }∥}^{2}$ to calculate the distance. Moreover, since the probability value obtained from the classification is based on the similarity of the distance between the class prototype vector and the feature vector, a smaller distance indicates higher similarity. Therefore, we need to invert the result of the distance function to reflect this relationship.

(14) $$p_{\theta }\left(y=k|x\right)=\frac{exp(-D\left(H,c_{k}\right))}{\sum_{i}exp\left(-D\left(H,c_{i}\right)\right)}$$

where D denotes the Euclidean distance. Finally, the training is optimized using Adam’s algorithm to minimize the negative logarithmic probability loss.

(15) $$J\left(\theta \right)=-logp_{\theta }\left(y=k|x\right)$$

## 4. Results

The software used in this research includes Windows 10, Pycharm Community 2023.1.2, Python 3.8.19, and PyTorch version 2.2.2+cu121. The hardware configurations consists of an i5-12600KF CPU, an NVIDIA GeForce RTX 4060 Ti GPU and 32G memory. The raw data used in this paper were collected from the shield advancement data of a tunnel project in Shenzhen, China, covering the period from 13:32 on 9 August 2022 to 0:48 on 11 October 2022. The data were obtained using the Herrenknecht large-diameter slurry pressure balance shield, which passed through geological conditions consisting of upper soft and lower hard composite strata (upper fully weathered mixed granite, middle earth/massive strongly weathered granite, and lower slightly weathered mixed granite), transitioning to hard rock strata (slightly weathered mixed granite). Since shield propulsion is conducted in rings, there is a significant stopping period between rings for assembling pipe pieces, with the digging process occupying only a small portion of the time. The wear process occurs almost exclusively during the digging phase when the disc cutter is rotating. Therefore, it is necessary to extract data corresponding to the digging working conditions during this period for analysis. After extraction, 393,887 working state data points were collected from the database. Due to sensor performance issues and failures, some data were duplicated or missing, necessitating de-duplication and interpolation. Subsequently, the data were sampled at 20-second intervals, resulting in 55,932 sequence data points after processing. Based on previous studies and observations of actual sensor data from the tunnel, 15 parameters were selected for analysis, and the details are provided in Table 2.

### 4.1. Engineering Background

Penetration indicates the distance advanced by the rotation of the disc cutter and serves as a measure of the overall cutting capacity of the disc. It is defined by P=v/f. *v* is the mean excavation speed, and *f* is the cutterhead speed. The FPI (Force per Penetration) reflects the positive force required during cutter boring, and the FPI is defined by FPI = F/P. *F* denotes the total thrust. The TPI (Tangential Force per Penetration) reflects the tangential force required during cutter boring, and the TPI is defined by TPI = T/P. *T* denotes the cutterhead torque.

In practical operations, wear amounts can only be measured during cutterhead inspections and cannot be collected in real time. Consequently, it is challenging to accurately determine the wear state labels of shield cutters based solely on wear measurements. To address this issue, we employed a combined approach using three methods to estimate the wear state:

(1) Empirical method: By consulting on site personnel and experts and combining data from periods of cutterhead inspection and downtime with the observed wear amount changes before and after inspections, the wear state intervals are estimated.
(2) Formula-Based Method: Using the wear calculation formula [37], the wear state intervals are deduced based on the amount of wear measured during cutter replacement. (This project defines an average wear amount of 10 mm as the threshold for the wear state). However, the method may be affected by abnormal wear, leading to inaccuracies in the estimation.
(3) Anomaly Detection Method: Based on related studies [38], this approach detects anomalies in shield operation. When an abnormal event occurs, the loss of the anomaly detection model increases significantly, providing a reference for identifying wear states. However, this method is also sensitive to other anomalies and therefore requires integration with empirical and formula-based methods for a comprehensive judgment.

Although none of these methods alone can determine the wear state with complete accuracy, combining these three approaches enables the most reliable estimation of the wear state range. Furthermore, through downsampling and feature engineering, the influence of a small number of mislabeled samples at state boundaries on the model’s detection accuracy can be minimized.

### 4.2. Data Preprocessing

**Figure 4:**
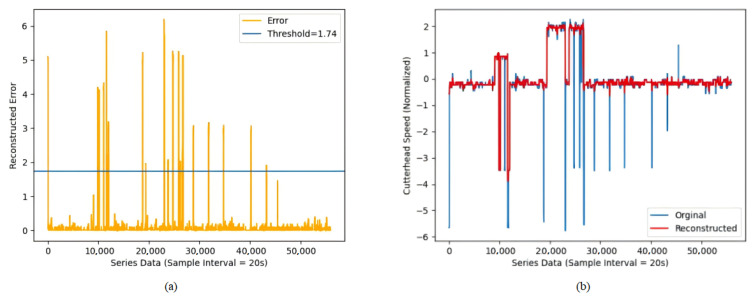
Outlier removal results for shield disc cutter speed. (**a**) The yellow curve represents the reconstruction bias of the LSTM-ED model, and the blue curve represents selected threshold value 1.74, which was calculated by 3σ criterion. (**b**) The blue curve represents the data before outlier removal; the red curve represents the data after outlier removal.

After that, the input data were normalized and divided into small sub-windows for reconstruction using a sliding window of length 15 and step size 1, resulting in a total of 55,917 windows. All the windows were input into the LSTM-ED model for training, where the LSTM hidden layer parameter was set to 64, and the number of layers was set to 1. The model was also optimized using the inverse-order reconstruction and teacher-forcing strategies. The cutterhead speed reconstruction results are shown in Figure 4, using the Adam optimizer training and setting the learning rate to 0.001.

**Figure 5:**
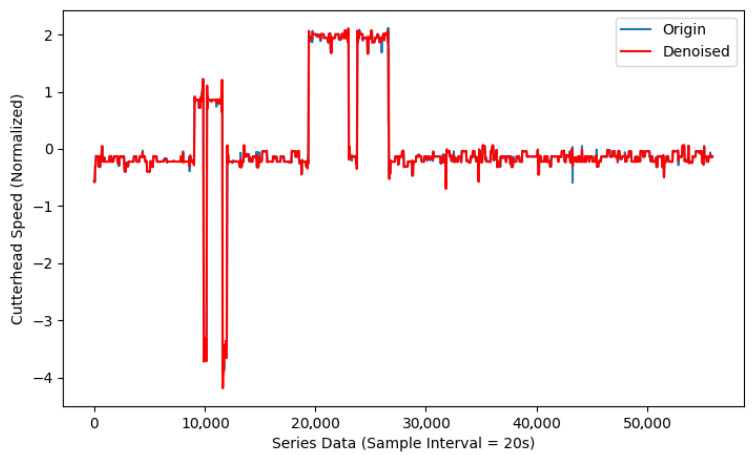
Denoising results of cutterhead speed using VMD-WT.

It can be seen that after filtering, the results appear significantly smoother, effectively addressing outliers and mitigating the impact of noise to a certain extent. In the next step, the data were denoised by VMD-WT transform. This paper decomposed the shield parameters into seven different IMFs, denoted as IMF1–IMF7, and set the penalty term to 2000, where IMF7 has the highest center frequency. IMF7 and the residuals of VMD decomposition could be eliminated due to the high-frequency noise and little contribution to the whole time series. Figure 5 shows the comparison results with cutterhead speed.

Finally, time-domain features were extracted separately for each parameter using sliding windows with a window length of 10 and a step size of 3. This process resulted in 165 primary features. Table 3 presents the results of calculating the trend and monotonicity scores for these features. Subsequently, the top 50 features with the highest scores were selected as model inputs for training.

**Figure 6:**
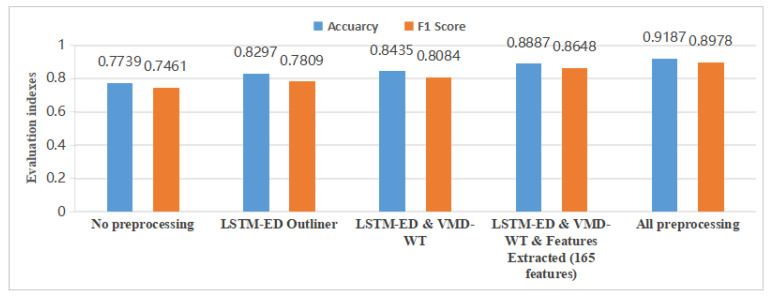
Effect of different preprocessing steps results with proposed model.

Since this paper employed various methods to preprocess the shield acquisition data, the impact of each preprocessing step on the results was evaluated by testing the data obtained at each stage using the proposed model. Figure 6 presents the final experimental results, where the outcomes of each step build upon the preceding step.

The results indicate that, compared to the initial feature selection without any preprocessing, the training performance improved by approximately 14% after preliminary data processing. This improvement is likely due to the limited wear data samples available for training, making it challenging to directly train the model to extract generalized disc cutter wear features. Additionally, it is observed that selecting only the top 50 features for training further enhanced performance by about 2% compared to using all features. This can be attributed to two factors: features outside the top 50 may have less impact on disc cutter wear detection, and excessively high-dimensional data can reduce computational efficiency and risk dimensionality explosion, ultimately affecting the model’s overall performance.

### 4.3. Comparison Models

After preprocessing, 18,668 trainable data were finally obtained. Considering the change in data distribution due to the gradual transition of the tunnel cut from the upper soft and lower hard strata to the hard rock strata during the acquisition of data for the segment, a total of 7348 pieces of data from one section of a complete upper soft and lower hard stratum and another section of a complete hard rock stratum were used as the training set, and the remaining 11,320 pieces of data were used as the test set for inspection. Six disc cutter wear events occurred in the shield and two in the selected training data. In the training dataset, the ratio of normal data to wear data is 6.5:1, which falls into the category of severe imbalance. Additionally, the total amount of training data is very small, consisting of only 7348 samples, making it a small-sample dataset. For the classification detection model hyperparameters, the learning rate LR was set to 0.0001, the batch size was set to 64, the number of iterations was 200, the sliding window length was 20, the LSTM output layer size was 128, the dropout layer was 0.2, the attention dimension was set to 64, the gradient clipping was set to 0.2, the loss function adopted the cross-entropy loss function, and we trained with the Adam optimizer.

The most commonly used metrics for evaluating classification results are accuracy and F1-score. In the case of shield data, where disc cutter wear abnormalities constitute a small proportion of the overall dataset and the dataset is inherently imbalanced, the F1-score provides a more representative measure of detection performance. Therefore, this paper adopted the F1-score as the primary evaluation metric for the model.

In order to show the advantages of the proposed model, we used important or newer deep learning models in the field of time series classification to compare, including (1) recurrent-based networks—LSTM-FCN, ALSTM-FCN [39], and BiLSTM; (2) kernel-based networks—ResNet and InceptionTime [40]; (3) transformer-based networks—GTN [41] and TARNet; and (4) other types of networks—TapNet [36]. Table 4 shows the experimental results.

The results indicate that our proposed model achieved superior performance on the shield disc cutter wear dataset compared to several prominent baseline methods, attaining an accuracy of 0.9187 and an F1-score of 0.8978, both of which surpassed the experimental outcomes of the other compared models. Notably, ALSTM-FCN outperformed BiLSTM, likely because ALSTM-FCN utilizes an attention mechanism to prioritize the importance of input features in shield data, which proves more significant than merely learning bi-directional dependencies within the data.

Additionally, the results reveal that the recurrent neural networks (RNNs) often performed comparably to, or even better than, the more complex kernel-based models on this dataset. This may be due to disc cutter wear data being more influenced by long-term time-dependent features, while kernel models tend to focus on numerical or shape-based features. This observation suggests that transformer-based network models, which excel at capturing long-range dependencies, could outperform both recurrent and kernel models. However, transformer models typically require large datasets for effective training, and acquiring sufficient wear data for disc cutter analysis remains challenging. This inherent data imbalance undermined the performance of the transformer-based models.

Interestingly, TapNet, which employs a prototype-like network structure, demonstrated comparable detection performance to the transformer models in real-world scenarios. The proposed MVSAPNet model builds upon these insights by incorporating a variable selection network to enhance feature selection capabilities. It also leverages the temporal dependency learning strengths of RNNs and adopts a prototype mechanism to address the dataset’s imbalance effectively. These design elements collectively enable MVSAPNet to deliver a better detection effect for disc cutter wear.

**Figure 7:**
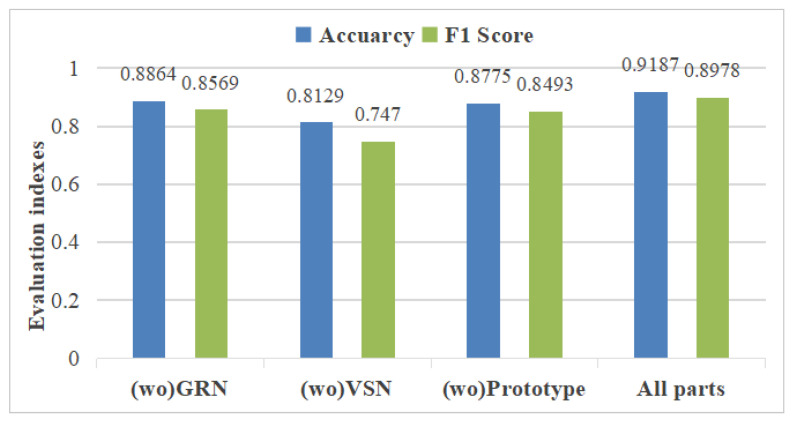
Impact of removing part of the network structure alone on detection performance.

In order to prove the effectiveness of each part of the proposed model for the whole network, ablation experiments were required, the results of which are shown in Figure 7. The GRN and VSN modules were directly removed, and the front and back inputs and outputs were directly connected. A fully connected layer replaced the prototype module. The results show that the GRN could better enhance the nonlinear ability of the model to learn the features of disc cutter wear better; the VSN could very effectively improve the detection effect of the model while selecting the variables through different weights and improve the interpretability of the disc wear anomalies to a certain extent by obtaining the selected weights. The prototype network could improve the model’s ability to detect disc cutter wear to a certain extent by using the attention mechanism to extract key features and the normal state as class prototype vectors and calculating the distance between the current state and the prototype vectors of shield for detection.

## 5. Discussion

**Figure 8:**
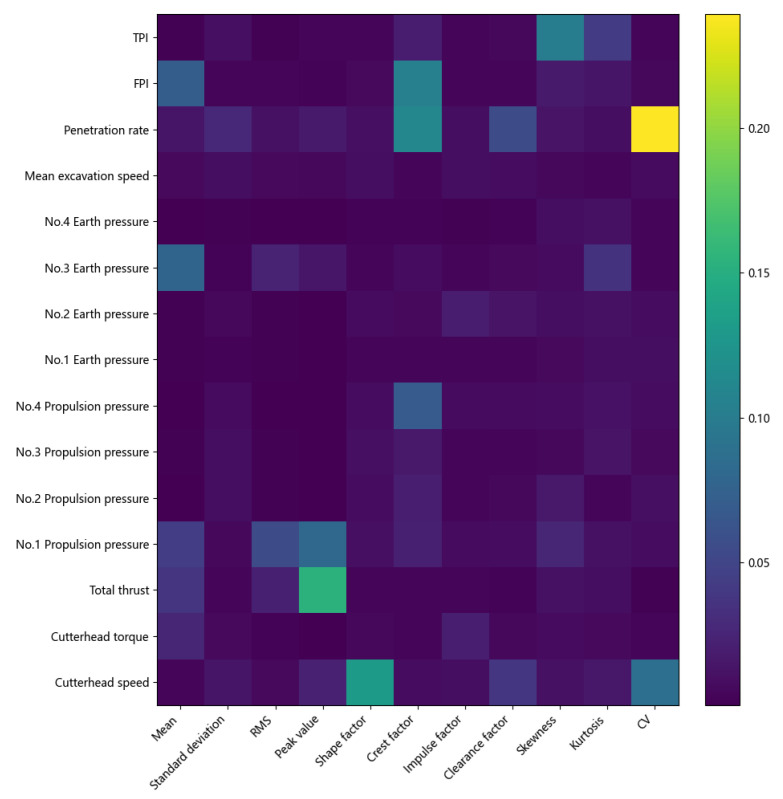
Visualization of mean weights of $v_{t}$ in VSNs.

To more accurately assess which shield parameters are more correlated with the overall level of disc cutter wear, we trained all features and visualized the selection weights $v_{t}$ in the variable selection module VSN, and the results are shown in Figure 8. In this figure, the horizontal axis represents the selected features. In contrast, the vertical axis represents the different sensor data captured by the shield, and the shade of the color is used to indicate the magnitude of the weights. The weights show that the variable selection network gave relatively large weights to the four parameters of TPI, FPI, penetration, and disc cutter speed in the composite strata. It indicates that when the overall disc cutter wear reached a certain level that affects the cutting efficiency and the cutter needs to be replaced, the four parameters of penetration, FPI, TPI, and cutterhead speed changed significantly. The weights of the other parameters are relatively small, particularly the cutterhead torque, which means the composite indicator parameters are more suitable for describing the shield state characteristics under different digging conditions than the single sensor parameters, reflecting the fact that a comprehensive judgment of the parameters is needed to judge the disc cutter wear, which is also the reason why evaluation indicators such as TPI, FPI, and penetration were proposed to be used for detecting the disc cutter wear in other works. In addition, it was also found that the excavation speed in the Ma Wan Cross-Sea Tunnel did not significantly impact the overall disc cutter wear. Actually, the data showed that the thrust in the sub-zones increased significantly when the disc cutter wear occurred. It is due to increasing the thrust on site to ensure excavation speed despite the disc cutter wear during construction. This finding is consistent with the summaries of experience and feedback reports from the engineers and technicians accumulated during the actual project.

**Figure 9:**
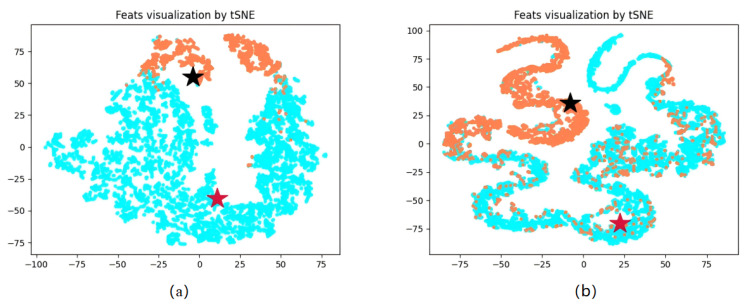
Visualization results of model sample features and class prototype feature using the t-SNE method, red and black stars for normal and wear state class prototype features, blue dots and orange dots for normal and wear state sample features. (**a**) Training set. (**b**) Test set.

In order to better visualize the relationship between individual samples in the model and the class prototype matrix, t-SNE was employed to project the class prototypes and the samples from both the training and testing phases. Figure 9 shows the results. In the figure, the orange points indicate the data of the disc cutter wear state, the blue points indicate the data of the normal state, and the black and red stars represent the centers of the class prototypes of the disc cutter wear state and the normal state. It can be seen that the distribution of the data features of the disc cutter wear state and the normal state has apparent differentiation. But some points were misclassified and scattered on the other side.

**Figure 10:**
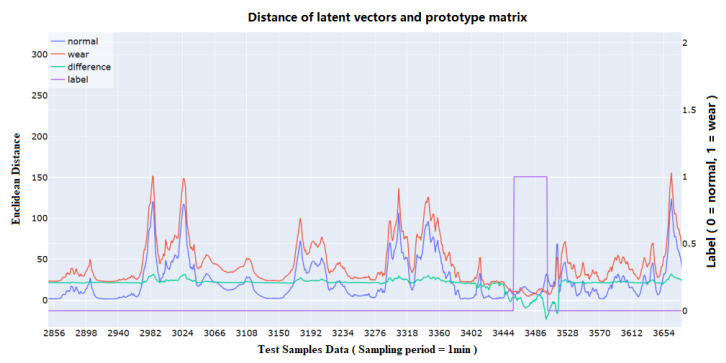
Visualization results of the distances between the sample vectors and the class prototype matrix on the test set of tunnel in Shenzhen, China, with the blue line being the distance between the samples and the normal state, the red line being the distance between the samples and the worn state, the green line being the worn distance and the normal distance, and the purple color being the class labels, with 0 = normal state and 1 = worn state.

We calculated the difference between the latent vectors and the different classes of vectors in the prototype matrix using the L2-norm to assess the changes in distances between the data features and the vectors of different classes during the advancement. Figure 10 shows the detection results of one of the cases in which disc cutter wear occurred. It can be found that when the shield’s working state is changed except for disc cutter wear, the distance between the shield and the different classes of prototypes will change simultaneously. In contrast, the distance relationship will change when the disc cutter wear occurs. By calculating the difference between the wear distance and normal distance, it can be more clearly seen that under normal propulsion, the difference is roughly stable even under different working conditions. However, when the cutting capacity of the disc cutter decreases, it means that the disc cutter needs to be changed. This difference will drop significantly, eventually reach a negative value, and eventually trigger an alarm. This can prove the model’s effectiveness in detecting disc cutter wear anomalies.

**Figure 11:**
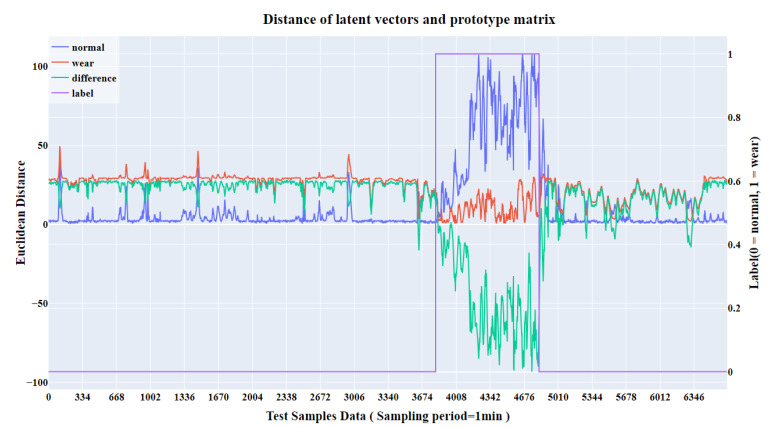
Visualization results of the distances in Fuzhou, China. Other is equal to Figure 10.

Additionally, we tested the model on a tunnel in Fuzhou, Fujian Province, China, using a large-diameter shield produced by Shanghai Tunnel Engineering Co., Ltd., and the results shown in Figure 11. After preprocessing, the test dataset consisted of 6691 samples, which included a segment with severe wear. The geological conditions in this area included a transition from blocky, strongly weathered granite porphyry to moderately weathered granite porphyry. To assess the model’s generalization and transferability, we tested it using the model trained on the Shenzhen tunnel dataset. Since the geological conditions of the two tunnels differ, we aligned the central distribution of the normal data from both the Fuzhou and Shenzhen tunnels. The results showed that with simple data adjustments, the model successfully detected shield cutter wear, achieving an accuracy of 0.9637 and an F1-score of 0.9347, demonstrating the model’s effectiveness and generalizability.

However, there are several limitations in this study. As observed from the t-SNE plot in Figure 9, although the model performed well on the training set, some data points in the test set were not well separated. This could be due to two reasons. First, the selected strata represent a composite transitional layer, and as the location changes, the distribution of wear states may drift. However, the training samples are continuous data without additional calibration or geological parameters to account for these variations. This lack of sensitivity to geological changes leads to misclassification at the edges of the wear interval. Second, as the exact wear values cannot be accurately measured, indirect methods introduce label errors, contaminating the data. This results in the model fitting to these errors during training, causing misclassifications. Additionally, as shown in Figure 10, the detection results exhibit some latency. While trends can be observed in advance for predictions, timely responses may not always be possible. It is also important to note that this study was conducted on a small, imbalanced dataset, with a 6.5:1 class ratio between positive and negative samples in the training set. Further testing on larger datasets is needed to validate the model’s continued effectiveness.

## 6. Conclusions

In this paper, we treated disc cutter wear detection as an end-to-end binary supervised classification problem and proposed a novel model called the Multivariate Selective Attention Prototype Network (MVSAPNet) for classification. Notably, we introduced the prototype network method for small-sample learning into the field of cutter wear detection for the first time while integrating a variable selection network to enhance the model’s interpretability. Due to the significantly smaller number of wear samples compared to normal samples in shield tunneling, conventional network training suffers from decreased performance. Existing methods to balance the data may lead to undertraining or ambiguous boundaries. In contrast, the proposed prototype network addresses these issues by learning a prototype for each class individually, alleviating data imbalance and making it well suited for small-sample training. Furthermore, by obtaining weights from the variable selection network, the model can explain the influence of input variables on the results. The proposed model was tested on historical data from a composite strata tunnel project in Shenzhen, achieving an accuracy of 0.9178 and an F1 score of 0.8978. These results outperform other advanced time series classification methods in this context, demonstrating the effectiveness of the approach. The model was also tested on a tunnel in Fuzhou, Fujian Province, and it was found that under similar conditions, cutter wear could be effectively detected with simple data alignment, demonstrating the model’s promising generalization and transferability.

Future work could focus on improving the feature extraction component to enhance its capability and address issues such as latency. Additionally, integrating geological parameters into the training process could help adapt to variations in different strata. Furthermore, testing on larger datasets is necessary to validate the model’s generalization ability and effectiveness.

## Figures and Tables

**Table 1:** Features selected for feature engineering.

Index	Feature	Equation	Index	Feature	Equation
1	Mean	$\bar{x}=\frac{1}{N}\sum_{i=1}^{N}x_{i}$	7	Impulse factor	$I=\frac{x_{p}}{\bar{x}}$
2	Standard deviation	$x_{\sigma }=\sqrt{\frac{1}{N-1}\sum_{i=1}^{N}{\left(x_{i}-\bar{x}\right)}^{2}}$	8	Clearance factor	$L=\frac{x_{p}}{{\left(\frac{1}{N}\sum_{i=1}^{N}\sqrt{|x_{i}|}\right)}^{2}}$
3	Root mean square	$x_{rms}=\sqrt{\frac{1}{N}\sum_{i=1}^{N}x_{i}^{2}}$	9	Skewness	$S=\frac{E[{\left(x-\bar{x}\right)}^{3}]}{x_{\sigma }^{3}}$
4	Peak value	$x_{p}=max\{|x_{i}\left|\right\}$	10	Kurtosis	$K=\frac{E[{\left(x-x\bar{x}\right)}^{4}]}{x_{\sigma }^{4}}$
5	Shape factor	$W=\frac{x_{rms}}{\bar{x}}$	11	CV	$CV=\frac{x_{\sigma }}{\bar{x}}$
6	Crest factor	$C=\frac{x_{p}}{x_{rms}}$			

**Table 2:** Details of 15 parameters selected.

Number	Parameter
1	Cutterhead speed (r/min)
2	Cutterhead torque (kN · m)
3	Total thrust (kN)
4–7	Propulsion pressure of cylinders groups No. 1–No. 4 (MPa)
8–11	Earth pressure of excavation soil bin No. 1–No. 4 (bar)
12	Mean excavation speed (mm/min)
13	Penetration (mm/r)
14	FPI
15	TPI

**Table 3:** Partial results of trend and monotonicity scores for features.

Features	Monotonicity	Trend	Score
Standard deviation of mean excavation speed	0.08	1	1.08
Kurtosis of cutterhead torque	0.1962	1.49 × $10^{-7}$	0.1962
Skewness of cutterhead torque	0.1962	0.0007	0.1781
Standard deviation of Earth pressure No. 1.	0.0171	0.1610	0.1781
…	…	…	…
Mean of Penetration	0.0952	0.0358	0.1310

**Table 4:** Performance comparison of different classification networks on test set of disc cutter wear.

Model	Accuarcy	F1-Score
LSTM-FCN	0.8151	0.7917
ALSTM-FCN	0.8427	0.8230
BiLSTM	0.8422	0.8172
ResNet	0.8385	0.8104
InceptionTime	0.8642	0.8412
TapNet	0.8594	0.8350
GTN	0.8848	0.8556
TARNet	0.9023	0.8785
MVSAPNet	0.9187	0.8978

## Data Availability

The data in the case study are not publicly available due to the confidentiality requirement of the project.
